# Supplementary material for: Tri-Phenyl-Phosphonium-Based Nano Vesicles: A New In Vitro Nanomolar-Active Weapon to Eradicate PLX-Resistant Melanoma Cells
Source: Int J Mol Sci. 2025 Mar 30;26(7):3227. doi: 10.3390/ijms26073227 (PMC11990052; doi:10.3390/ijms26073227)
Supplement: Supplementary file 1 [file ijms-26-03227-s001.zip › ijms-3522412-supplementary.pdf]

International Journal of *Molecular Sciences*

MDPI

Supplementary Materials

Tri-Phenyl-Phosphonium-Based Nano Vesicles: A New In Vitro Nanomolar-Active Weapon to Eradicate PLX-Resistant Melanoma Cells

Silvana Alfei [1,*], Carola Torazza [1], Francesca Bacchetti [1], Maria Grazia Signorello [2], Mario Passalacqua [3,4], Cinzia Domenicotti [4,5,6] and Barbara Marengo [4,5,6,*]

[1] Department of Pharmacy, University of Genoa, Viale Cembrano, 16148 Genoa, Italy; carola.torazza@unige.it (C.T.), francesca.bacchetti@edu.unige.it (F.B.)

[2] Biochemistry Laboratory, Department of Pharmacy, University of Genoa, Viale Benedetto XV 3, 16132 Genova, Italy; mariagrazia.signorello@unige.it

[3] Biochemistry Section, Department of Experimental Medicine (DIMES), University of Genova, Via Alberti L.B., 16132 Genoa, Italy; mario.passalacqua@unige.it

[4] Centro 3R, Department of Information Engineering, University of Pisa, Largo Lucio Lazzarino 1, 56122 Pisa, Italy; cinzia.domenicotti@unige.it

[5] IRCCS Ospedale Policlinico San Martino, 16132 Genova, Italy

[6] Department of Experimental Medicine (DIMES), University of Genova, Via Alberti L.B., 16132 Genoa, Italy

* Correspondence: alfei@difar.unige.it (S.A.); barbara.marengo@unige.it (B.M.); Tel.: +39-010-355-2296 (S.A.)

Section S1: Excel Graphs

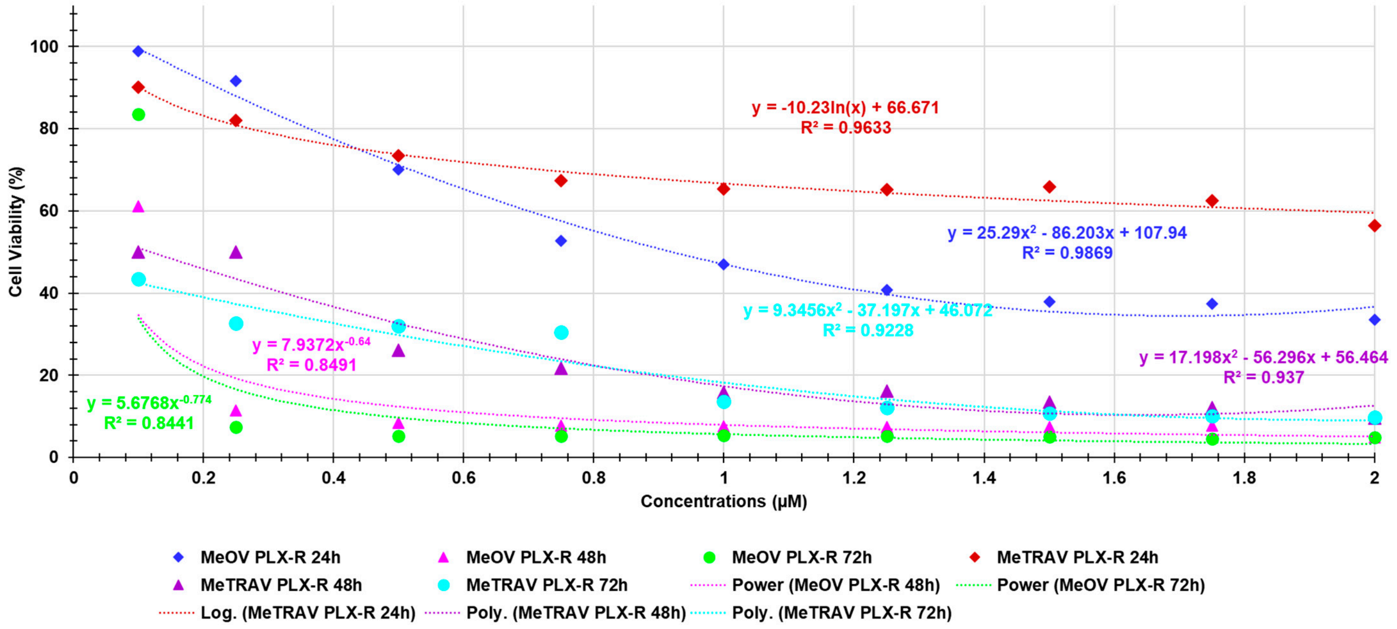

Figure S1. Dispersion graphs (no lines) of cell viability (%) of PLX-R MeOV and MeTARV cells vs. increasing BPPB concentrations (0.1-2.0 µM) after 24 hours (blue and red square indicators, respectively), 48 hours (pink and purple triangular indicators, respectively) and 72 hours (green and sky-blue spherical indicators, respectively) of exposure. Dotted lines represent the nonlinear regression models which best fitted the data of dispersion graphs, according to R^2. In same colours, the associated R-squared values and the equations expressing the mathematical relationship existing between data, have been shown.

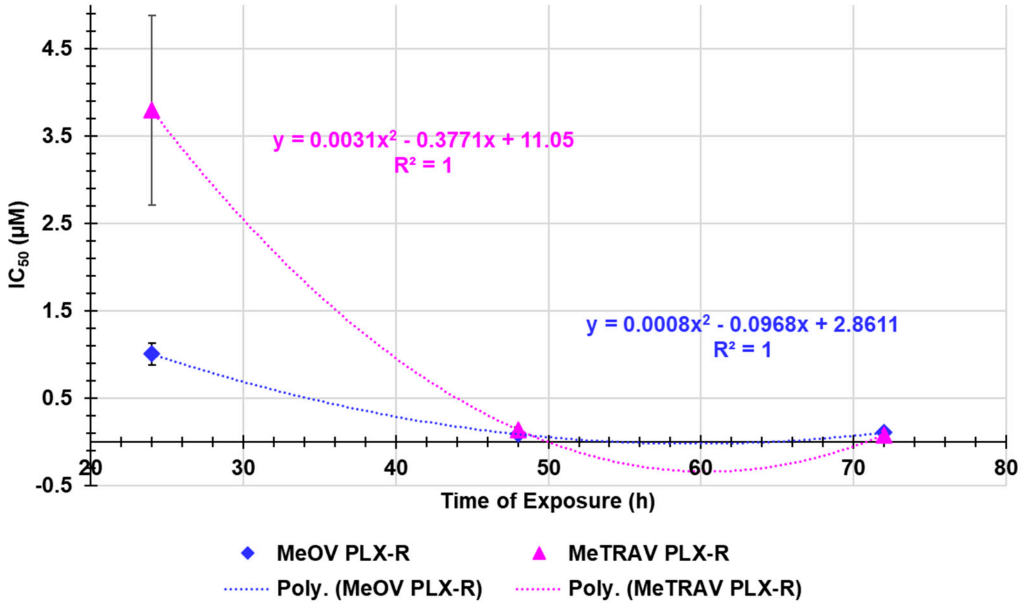

Figure S2. Dispersion graphs of IC_{50} values of BPPB towards PLX-R MeOV (blue square indicators) and PLX-R MeTRAV (pink triangular indicators) cells as functions of exposure timing with related second order polynomial regression models (punctuated blue and pink lines), their equations and R^2 values, as provided by Microsoft Excel 365 software. The IC_{50} value of BPPB on PLX-R MeTRAV cells at 24 hours of exposure is an extrapolation predicted by the nonlinear model constructed by using GraphPad Prism 8.0.1 Software (GraphPad Software, Boston, MA, USA).

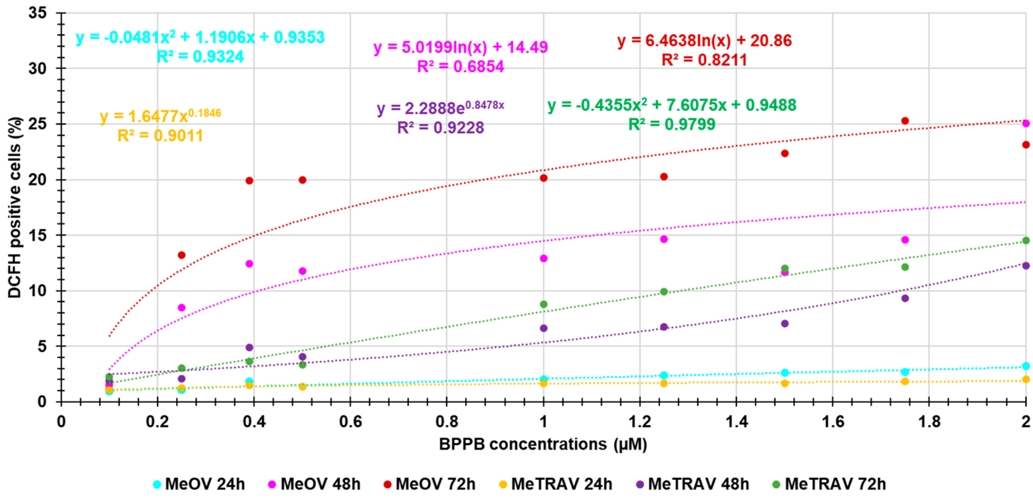

Figure S3. Dispersion graphs of the values of DCFH positive cells (%) vs. BPPB concentrations (µM) concerning PLX-R MeOV and MeTRAV cells after 24 (sky-blue and yellow round indicators, respectively) 48 (pink and purple round indicators, respectively) and 72 hours (red and green round indicators, respectively) of treatment. Punctuated lines with the same colors of indicators represent the best fitting nonlinear statistical models, which have been shown with their equations and R^2 values.

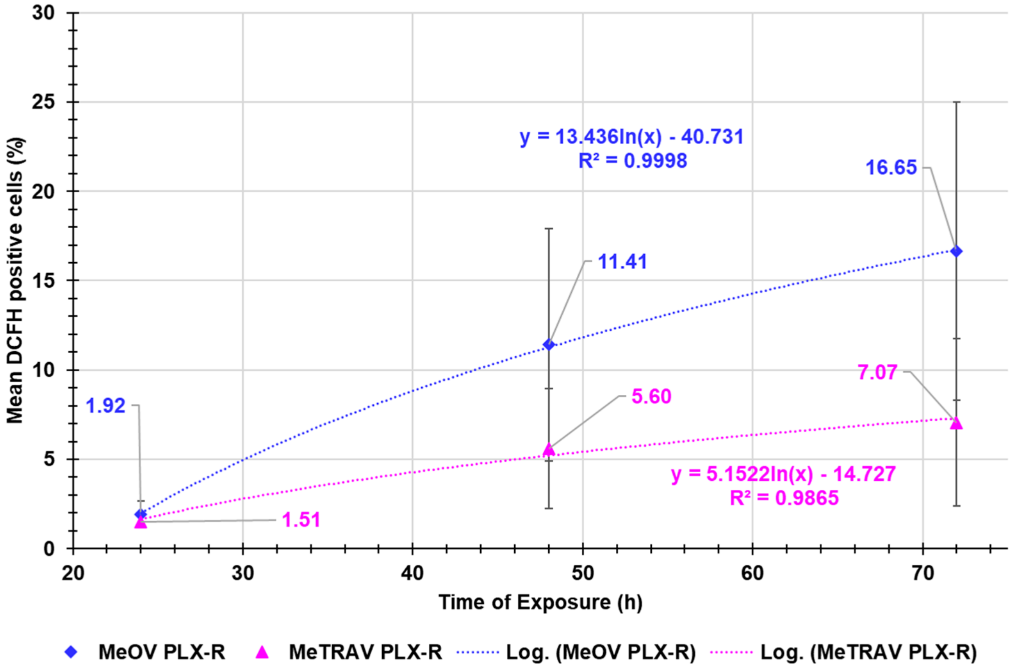

Figure S4. Dispersion graphs of the average DCFH positive cells (%) observed for both cells' lines vs. exposure timing, with the related nonlinear regression models which best fitted the data and their R^2 values.

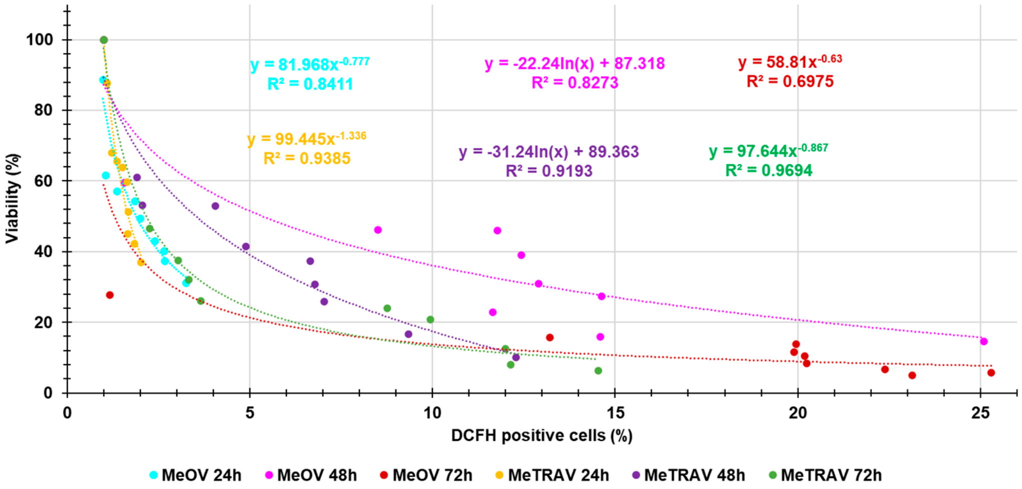

Figure S5. Dispersion graphs of the values of DCFH positive cells (%) vs. cells viability concerning PLX-R MeOV and MeTRAV cells after 24 (sky-blue and yellow round indicators, respectively) 48 (pink and purple round indicators, respectively) and 72 hours (red and green round indicators, respectively) of treatment. Punctuated lines with the same colors of indicators represent the best fitting nonlinear statistical models, which have been shown with their equations and R^2 values.

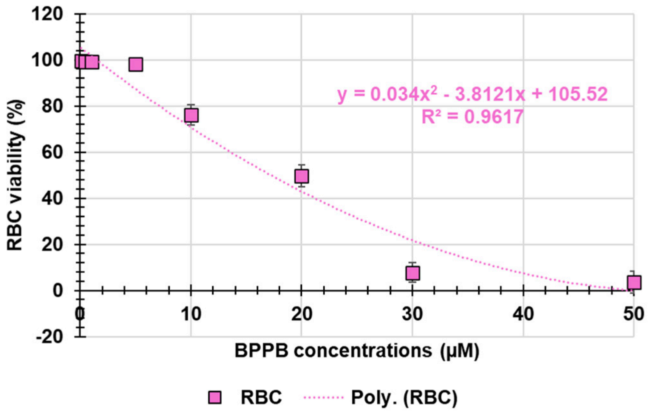

Figure S6. Dispersion graph of the RBCs viability (%) vs. BPPB concentrations in the range 0.1-50 μM, the related best fitting nonlinear statistical model with its equation and R^2 value.

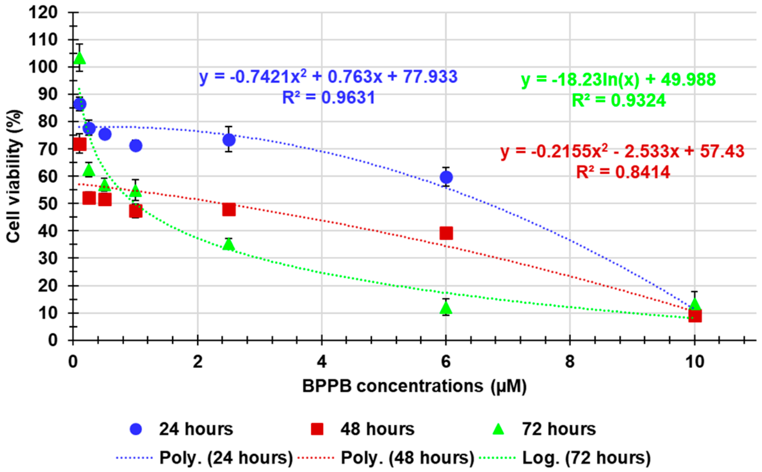

Figure S7. Dispersion graphs of the HaCaT cells viability (%) vs. BPPB concentrations in the range 0.1-10.0 μM after 24-, 48- and 72-hours treatments, the related best fitting nonlinear statistical models with their equations and R^2 value.

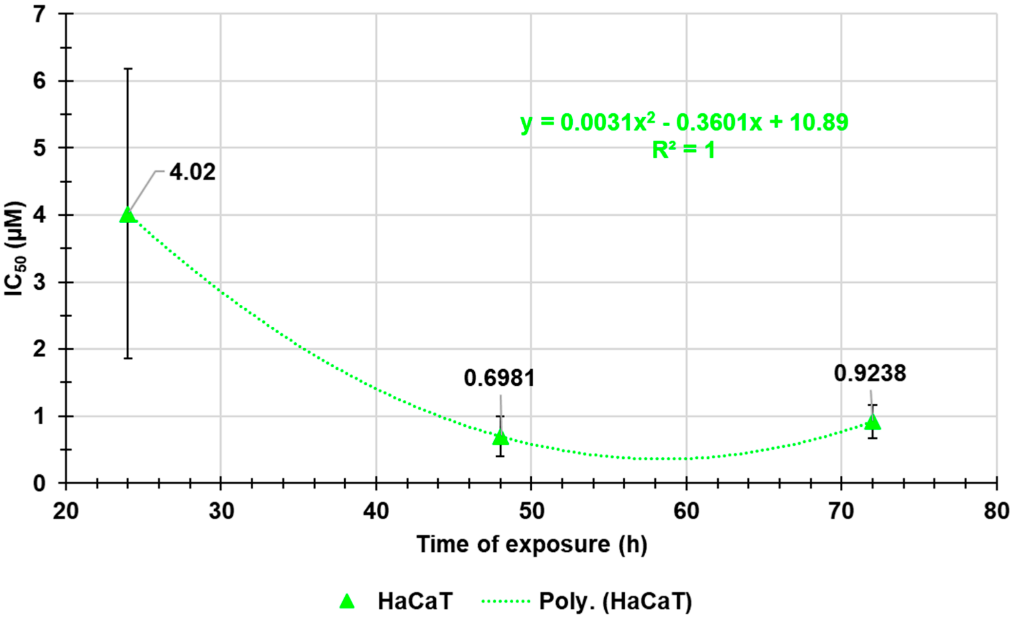

Figure S8. Dispersion graphs of IC_{50} values of BPPB towards HaCaT cells (green triangular indicators) as functions of exposure timing with related second order polynomial regression model (punctuated green line), its equation and R^2 value, as provided by Microsoft Excel 365 software.

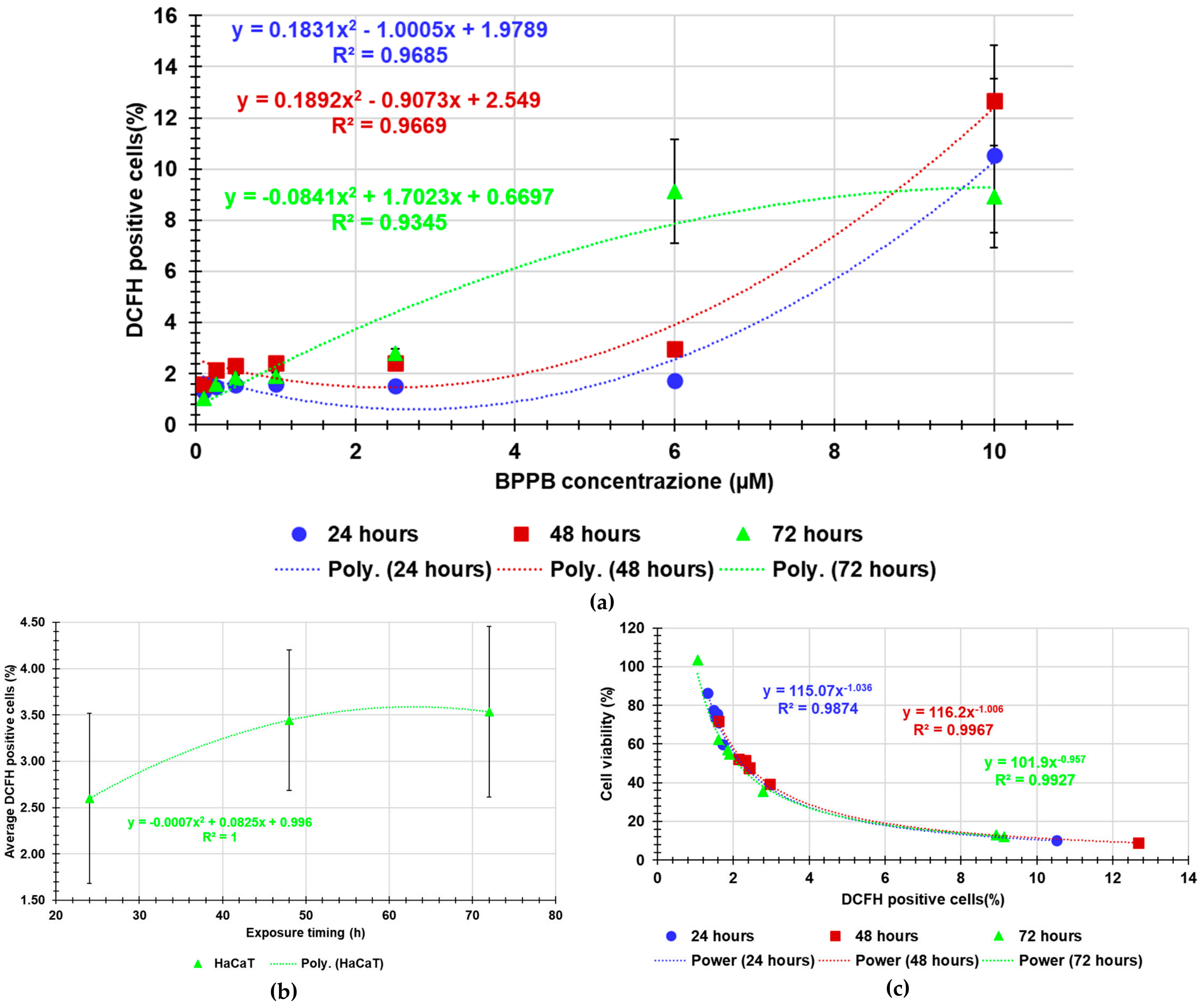

Figure S9. Dispersion graphs of the DCFH positive cells (%) into HaCaT cells vs. BPPB concentrations in the range 0.1-10 µM, after 24-, 48- and 72-hours treatments (a). Dispersion graphs of the average DCFH positive cells (%) into HaCaT vs. exposure timings (green triangular indicators with error bars) (b). Dispersion graphs of HaCaT cell viability (%) vs. the DCFH positive cells (%) into HaCaT cells after exposure to increasing concentrations (0.1-10.0 µM) of BPPB for 24 (round blue indicators), 48 (square red indicators) and 72 (triangular green indicators) hours(c). The dotted lines in Figure S9a, S9b and S9c in the same colors of indicators represent the best fitting nonlinear models which have been reported with their equations and R^2 values, as provided by Microsoft Excel 365 software.

Section S2: Tables

Table S1. IC_{50} values of BPPB on Cos-7 and HepG2 cells measured from the results of cytotoxicity carried out in the range of concentrations of 0.4–85.3 µM.

Immortalized Cells	IC_{50} 24 h (µM)
Cos-7 *	4.9100 ± 0.8100
HepG2 **	9.6400 ± 1.3100

* Monkey kidney cells; ** human liver cells.

Table S2. IC_{50} values of BPPB on MRC-5 cells measured from results of cytotoxicity carried out in the range of concentrations of 0.5–5 μM.

Exposure Time (hours)	IC_{50} MRC-5 (μM)
24	2.7740 ± 2.6655
48	0.7395 ± 0.5716
72	0.9277 ± 0.8956
